# The State of Artificial Intelligence in Nursing Education: Past, Present, and Future Directions

**DOI:** 10.3390/ijerph20064884

**Published:** 2023-03-10

**Authors:** Jennie C. De Gagne

**Affiliations:** School of Nursing, Duke University, 307 Trent Drive, DUMC 3322, Durham, NC 27710, USA; jennie.degagne@duke.edu

## 1. Introduction

As health care continues to evolve and become increasingly complex, nursing education must also evolve to keep pace with the changing landscape. One significant development in higher education has been the integration of artificial intelligence (AI) technology, which has the potential to transform education by providing more personalized and efficient learning experiences for students [1]. As with any new technology, concerns and controversies surround the adoption of AI into higher education, including nursing programs [2]. This article aims to provide an overview of the state of AI in nursing education by examining its historical roots, current applications, and future directions. By discussing the opportunities and limitations of AI in nursing education, this article encourages nurse educators to reflect on how best to integrate AI technology into their teaching to enhance student learning and contribute to the development of competent and compassionate nurses.

## 2. AI’s Roots, Growth, and Benefits

The integration of AI technology into higher education has a long history that dates back to the 1950s, when it emerged as a niche area of research with limited interest [3]. AI gained popularity in the 1970s and 1980s due to the development of modern computing technology [1]. In the 1960s, researchers investigated the use of computer-assisted instruction, and by the late 1960s, natural language processing had begun, which improved through self-play and was one of the first instances of a working machine learning system [4]. The use of computer-assisted instruction expanded in the 1970s, resulting in the creation of early computer-based teaching materials, such as multimedia learning resources, interactive simulations, and online tutorials, that demonstrated the potential of AI to improve experiences of teaching and learning [1]. In the 1990s, the use of AI-generated data through learning analytics and intelligent tutoring systems was introduced and demonstrated improved student performance [1]. These early experiments made it possible to provide personalized learning experiences and active learning [5]. A recent example of AI in nursing education is ChatGPT, which can generate a variety of mock simulation cases, such as patient interviews or job interviews, providing interactive learning experiences while saving educators’ time. AI can also automate assessment and grading, allowing nursing faculty to focus on other aspects of teaching [2].

The use of AI in higher education has garnered global attention, with several countries investing in AI research and education. China has launched a national AI development plan to achieve significant progress in AI research and innovation by 2030 [6]. South Korea is dedicating significant resources to developing AI education and research with the goal of building human capacity and anticipating labor market shifts; to cultivate AI talent, the country is taking measures such as increasing the number of AI graduate schools and offering short-term intensive educational programs [7]. The European Union’s Digital Education Action Plan 2021–2027 aims to enhance student learning and support teachers and administrative staff by promoting the use of AI in education [8]. The National Science Foundation in the United States is also investing in AI education and research, with a focus on improving equity in education through the use of AI-augmented learning for adult learners [9]. The investment in AI education and research by countries worldwide is aimed at developing leadership in the field and preparing students for the future workforce.

AI technology holds significant potential to create more sophisticated and complex simulations that can help nursing students develop critical thinking skills and prepare for real-world patient care situations. Such simulations can provide students with realistic scenarios that mimic patient care situations, allowing them to practice their clinical skills and decision making in a safe environment. As AI technology advances, these simulations will become even more advanced and sophisticated, offering an increasingly realistic and immersive learning experience. The benefits of AI in nursing education, such as interactive learning experiences and time-saving opportunities, are undeniable, but potential risks necessitate a cautious and informed approach to its use. For instance, the use of ChatGPT, an AI chatbot system, in nursing education has elicited concerns about breaches of academic integrity and ethics and theft of intellectual property [2]. When implemented according to proper guidelines and used ethically, however, AI can significantly improve learning experiences for nursing students and better prepare them for the challenges of a rapidly changing health care landscape.

## 3. Navigating the Opportunities and Challenges of AI in Nursing Education

The integration of AI into nursing education presents a wide range of opportunities, including enhanced learning outcomes and improved efficiency; however, it also poses several challenges related to privacy and security, ethical considerations, and resistance to adoption. A primary challenge associated with AI integration is the protection of student privacy. Given that AI requires access to personal information, it is essential to maintain student confidentiality and safeguard against any potential data breaches that could compromise student privacy. Ethical issues related to data bias, the exacerbation of existing inequalities, and adherence to professional standards must be thoughtfully considered and thoroughly addressed. To address such concerns, it is essential to promote ethical AI practices, provide appropriate training and support to educators and students, and implement robust privacy and security measures.

As with any new technology, resistance to the adoption of AI-based tools and techniques is a challenge that nursing education programs will likely face. Faculty members may have concerns about the potential impact of AI on their workload or their role in the teaching process; some may fear that they will need to expend a significant amount of time learning how to use AI tools or that AI could replace human educators altogether. In addressing these concerns, it is important to note that AI technology supplements and enhances the teaching and learning process when used correctly. AI-based tools can help to streamline administrative tasks and provide personalized learning experiences that cater to the unique needs of individual students. By automating routine tasks, such as grading, attendance monitoring, and student progress tracking, nurse educators gain time to focus their efforts on more complex teaching tasks that require their unique insight and expertise.

Although AI-based tools and techniques can offer significant benefits to nursing education, it is crucial to ensure that they supplement and enhance, rather than replace, human interaction, critical thinking, and creativity. The role of nurse educators in fostering these essential skills is critical to the development of competent and compassionate nurses. By striking a balance between AI-powered tools and human interaction, nursing schools can provide a more holistic and effective learning experience for students. Collaborative efforts between AI researchers and nurse educators can lead to a comprehensive and nuanced approach to nursing education that leverages the benefits of technology while preserving the value of human interaction.

To address concerns about workload and the impact of AI on their teaching role, nurse educators must receive proper training and support. Training should be designed specifically to help faculty understand the capabilities and limitations of AI technology and how it can be used to enhance the teaching and learning process. Nurse educators should be provided with guidance on how to use AI-based tools effectively and integrate them into existing teaching practices to make the most of their potential. Moreover, nurse educators can play a critical role in developing and designing AI-powered tools that align with the goals of nursing education and the core values of the nursing profession. Nurse educators can provide valuable input on the features and functionalities they consider most important, and they can ensure that tools are designed to support clinical judgment and patient-centered care.

Implementing AI-based tools in the nursing curriculum can involve significant costs and may require investments in technology and infrastructure to support their integration, such as expenses related to software, hardware, and staff training. However, by providing proper training and technical support, while involving instructional design experts and nurse educators in the development of a technology-enhanced curriculum, nursing education programs can effectively address concerns and ensure that AI is integrated responsibly and ethically in accordance with the core values of nursing education. One way to do this is by reallocating resources from existing budgets, while seeking external support for grants or collaborating with industry partners. 

Investing in AI-based tools and techniques can yield long-term benefits for nursing education, such as improved learning outcomes, enhanced efficiency, increased effectiveness of teaching practices [5]. These benefits ultimately produce competent and compassionate nurses who are well equipped to provide high-quality care in a rapidly evolving health care landscape. Collaboration between instructional design experts, nurse educators, and AI researchers can lead to a comprehensive and nuanced approach to nursing education that leverages the benefits of technology while preserving the value of human interaction. Securing adequate funding is crucial for nursing education programs to remain at the forefront of innovation and to provide nursing students with access to some of the most up-to-date and effective learning experiences possible.

## 4. Conclusions

The integration of AI technology in nursing education has the potential to revolutionize by providing personalized learning experiences and improving efficiency and outcomes. However, the ethical and responsible use of AI must be ensured through careful consideration and effective strategies that address concerns such as privacy, security, bias, and adherence to professional standards. Furthermore, continued research and innovation in the field of AI in nursing education will be crucial to exploring the best practices for incorporating AI technology, examining its impact on student learning and program outcomes, and addressing ethical and legal concerns. With proper implementation and guidelines, AI tools can complement and enhance human interactions in nursing education, preparing nursing students for a rapidly changing health care landscape and advancing the nursing profession.
